# Lymphatic dysfunction attenuates tumor immunity through impaired antigen presentation

**DOI:** 10.18632/oncotarget.4018

**Published:** 2015-05-27

**Authors:** Takayuki Kimura, Makoto Sugaya, Tomonori Oka, Andrew Blauvelt, Hitoshi Okochi, Shinichi Sato

**Affiliations:** ^1^ Department of Dermatology, Faculty of Medicine, University of Tokyo, Tokyo, Japan; ^2^ Department of Regenerative Medicine, Research Institute, National Center for Global Health and Medicine, Tokyo, Japan; ^3^ Oregon Medical Research Center, Portland, Oregan, USA

**Keywords:** lymphatics, lymphedema, tumor immunity, cytotoxic T cells

## Abstract

Tumor growth and metastasis of cancer involve autonomous tumor cell growth and host-tumor interactions. While tumor-specific immunity has been intensively studied *in vitro*, dynamic roles of lymphatic transport on tumor immunity *in vivo* have not been fully elucidated. In this study, we examined tumor growth and anti-tumor immune responses using *kCYC* mice, which demonstrate severe lymphatic dysfunction. Primary tumor growth was augmented in *kCYC* mice (compared to wild-type mice) when B16 melanoma or EL-4 lymphoma cells were subcutaneously injected. Expression of inflammatory cytokines such as IFN-γ, TNF-α, and IL-2 as well as IL-10 expression in draining lymph nodes (LNs) was significantly reduced in *kCYC* mice after tumor inoculation. Moreover, decreased levels of tumor-associated antigens were detected in draining LNs in *kCYC* mice, together with impaired antigen presentation. CD8^+^ T cells in draining LNs derived from *kCYC* mice bearing B16 melanoma also showed significantly decreased cytotoxic activity *in vitro*. Finally, tumor suppression activity of CD8^+^ T cells derived from *kCYC* mice bearing B16 melanoma was reduced when adoptively transferred to naive wild-type mice. In summary, these findings suggest that lymphatic transport is essential in generating optimal tumor-specific immune responses mediated by CD8^+^ T cells.

## INTRODUCTION

Tumor growth and metastasis of cancer involve autonomous tumor cell growth and host–tumor interactions [1]. Immune cells with predominantly anti-tumor functionality include cells of the innate immune system, such as natural killer (NK) cells [2], and cells of adaptive immunity, such as dendritic cells (DCs) [3] and CD8^+^ T cells [4]. Although function of each of these cell types in tumor immunity has been examined, anti-tumor activity of immune cells in the context of lymphatic dysfunction is yet to be fully elucidated.

The lymphatic system collects extravasated fluid, macromolecules, and cells of the immune system within tissues and returns them to the blood circulation [5]. Lymphedema occurs when there is interstitial accumulation of protein-rich fluid and subsequent inflammation, adipose tissue hypertrophy, and fibrosis. Recently, more attention has been paid to this condition since it is a relatively common complication following surgical treatment of malignancy [6]. Lymphedema is associated with a number of complications, including infections with bacteria and fungi. In rare cases, patients with lymphedema develop angiosarcoma [7, 8], squamous cell carcinoma [9, 10], or lymphoma [11, 12]. Reduced tissue immune surveillance secondary to lymphatic dysfunction may cause cancer. Indeed, lymphatic vessels are critical for transporting tissue-resident DCs and other immune cells as well as interstitial fluid to the lymph nodes (LNs), which is important in immunity against infectious agents and malignancy. Recently the role of lymphatic drainage on local immunity has been receiving attention, although only few studies were performed using mouse models [13–18].

Specific markers for lymphatic endothelium include vascular endothelial growth factor receptor (VEGFR)-3 [19, 20], podoplanin [21], and LYVE-1 [22]. We generated transgenic mice expressing the Kaposi's sarcoma-associated herpesvirus latent-cycle gene, *k-cyclin*, under the control of theVEGFR-3 promoter [23]. In Kaposi's sarcoma, this viral gene is expressed by lymphatic endothelial cells and probably contributes to edema within tumors [24, 25]. Interestingly, most *k-cyclin* transgenic (*kCYC^+/–^*) mice develop progressive accumulation of chylous pleural fluid. In skin, extensive dermal edema is detected by magnetic resonance imaging [23]. In addition, lymphatic drainage of injected contrast dyes is markedly impaired in these transgenic mice. In this study, we utilized these mice to investigate the role of the lymphatic system in tumor immunity. We have revealed, in the setting of lymphatic dysfunction, that tumor growth is significantly increased due to impaired tumor immunity.

## RESULTS

### Increased primary tumor growth of B16 melanoma cells in *kCYC* mice

**Figure 1:**
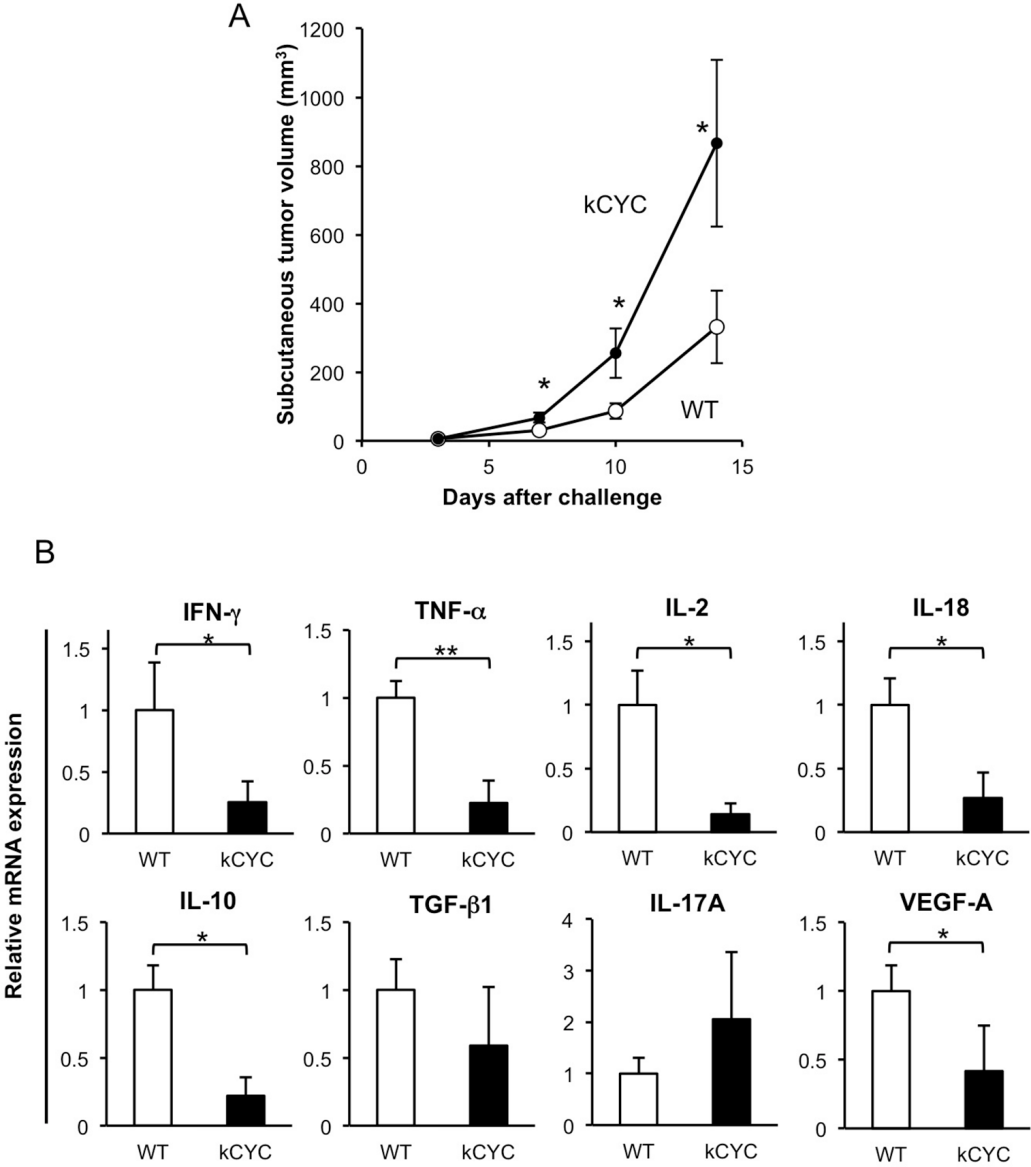
Primary tumor growth of B16 melanoma cells is promoted, while cytokine expression in draining LNs is decreased in *kCYC* mice **A.** Tumor volume was measured at the indicated time points following subcutaneous injection of B16 melanoma cells. Values represent means ± SEM (*p < 0.01). These results were obtained from 22 mice in each group. **B.** Cytokine mRNA expression in draining LNs from *kCYC* mice and wild-type (WT) mice 14 days following subcutaneous injection of B16 melanoma cells. mRNA expression of interferon (IFN)-γ, tumor necrosis factor (TNF)-α, interleukin (IL)-2, IL-18, IL-10, transforming growth factor (TGF)-β, IL-17A, vascular endothelial growth factor (VEGF)-A was measured by quantitative PCR. All values represent means + SEM (*p < 0.05, **p < 0.02). Results were obtained from 6 mice in each group.

Subcutaneous injection of B16 melanoma was used as a model of primary tumorigenesis by establishing a focus of these cells near the skin, the site of origin for melanoma [26]. To evaluate the effects of lymphatic dysfunction on primary tumor growth, B16 melanoma cells were injected subcutaneously into *kCYC* and wild-type (WT) mice, and tumor growth was determined on days 3, 7, 10, and 14. On days 7, 10, and 14 the tumor volume in *kCYC* mice was significantly greater than that in WT mice (Figure 1A, *p* < 0.01). Thus, primary tumor growth of melanoma cells was augmented in the setting of lymphatic dysfunction.

### Cytokine expression in draining LNs is decreased in *kCYC* mice following subcutaneous injection of B16 melanoma cells

Pro-inflammatory cytokines, including IFN-γ, TNF-α, and IL-2 are critical in tumor initiation, promotion, and progression. To assess the effect of lymphatic dysfunction on cytokine release during tumor development, expression levels of cytokines were measured in draining LNs 14 days following tumor inoculation in *kCYC* and WT mice. Messenger RNA levels of IFN-γ, TNF-α, IL-2, and IL-18 were significantly decreased in *kCYC* mice compared with WT mice on day 14 (Figure 1B, *p* < 0.05, 0.02, 0.05, and 0.05, respectively). Interestingly, IL-10 expression was also reduced in *kCYC* mice (*p* < 0.05), however, messenger RNA levels of TGF-β and IL-17A were not significantly different between these two groups. Expression levels of vascular endothelial growth factor (VEGF)-A, which mediates angiogenesis and drives inflammatory process [27, 28], were significantly lower in *kCYC* mice than in WT mice (*p* < 0.05). Thus, in the setting of lymphatic dysfunction, expression of inflammatory cytokines as well as IL-10 was decreased in draining LNs following subcutaneous injection of B16 melanoma cells.

### Primary tumor growth is promoted, while cytokine expression in draining LNs is decreased, in *kCYC* mice following subcutaneous injection of EL4 lymphoma cells

**Figure 2:**
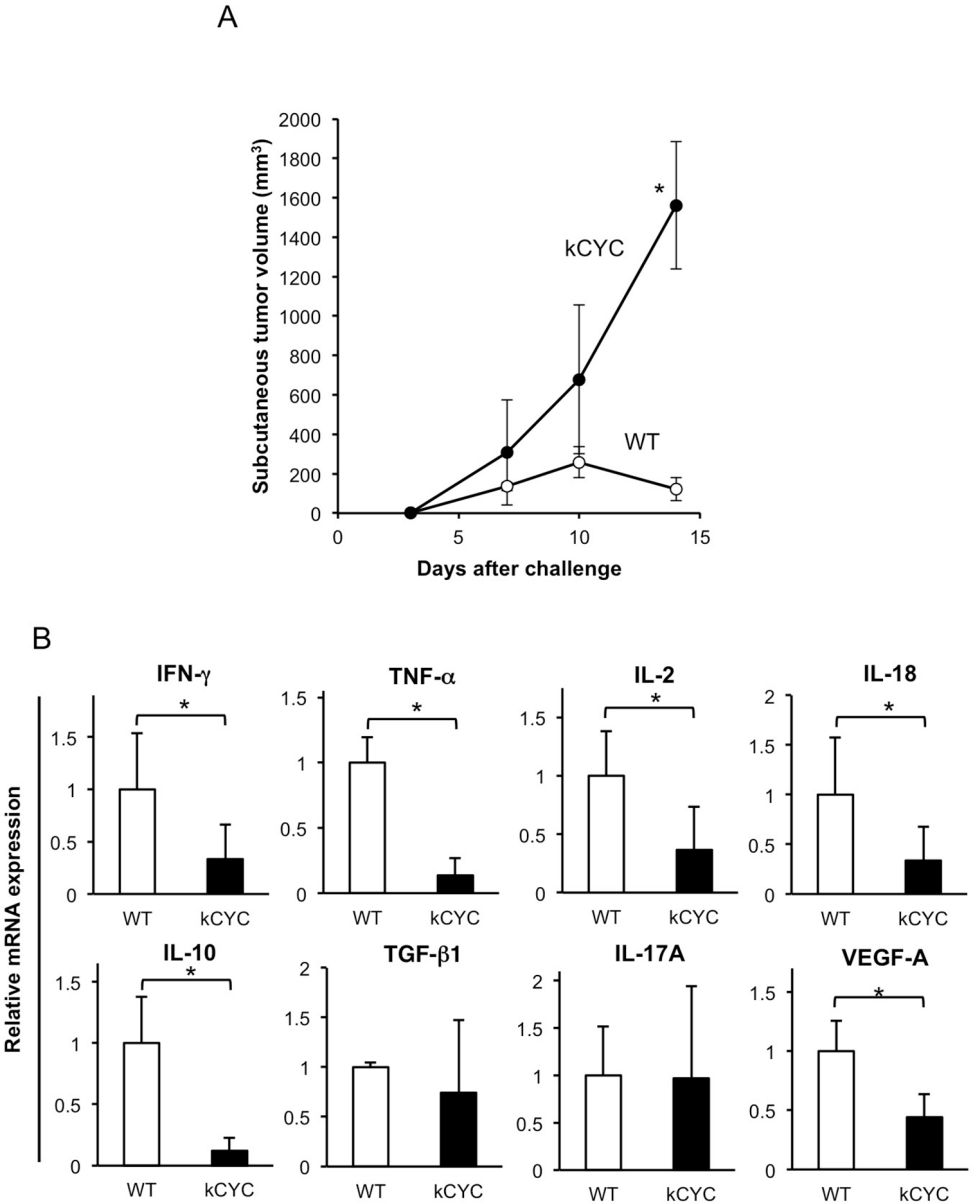
Primary tumor growth of EL-4 lymphoma cells is promoted, while cytokine expression in draining LNs is decreased in *kCYC* mice **A.** Tumor volume was measured at the indicated time points following subcutaneous injection of EL-4 cells. Values represent means ± SEM (*p < 0.05). Results were obtained from 4 mice in each group. **B.** Cytokine mRNA expression in draining LNs from *kCYC* mice and wild-type (WT) mice 14 days following subcutaneous injection of EL-4 cells. mRNA expression of interferon (IFN)-γ, tumor necrosis factor (TNF)-α, interleukin (IL)-2, IL-18, IL-10, transforming growth factor (TGF)-β, IL-17A, vascular endothelial growth factor (VEGF)-A was measured by quantitative PCR. All values represent means + SEM (*p < 0.05). Results were obtained from 4 mice in each group.

Subcutaneous injection of EL-4 lymphoma was used as an additional model of primary tumorigenesis. To evaluate the effects of lymphatic dysfunction on primary tumor growth, EL-4 lymphoma cells were injected subcutaneously into *kCYC* and WT mice in the same manner as B16 melanoma cells, and tumor growth was determined on days 3, 7, 10, and 14. Skin tumors in *kCYC* mice tended to be larger than in WT mice on days 7 and 10, but did not reach statistical significance. By day 14, however, lymphoma tumor volumes in *kCYC* mice were significantly greater than that in WT mice (Figure 2A, *p* < 0.05). As shown with melanoma, mRNA levels of IFN-γ, TNF-α, IL-2, IL-18, IL-10, and VEGF-A were significantly decreased in *kCYC* mice compared with WT mice in draining LNs on day 14 (Figure 2B, *p* < 0.05, respectively). TGF-β and IL-17A mRNA levels were not significantly different between these two groups. Thus, primary tumor growth was augmented, while cytokine expression in draining LNs was decreased, in the setting of lymphatic dysfunction, regardless of the tumor cell origin.

### Less frequent tumor metastasis and decreased levels of tumor antigens in draining LNs of *kCYC* mice compared to WT mice

**Figure 3:**
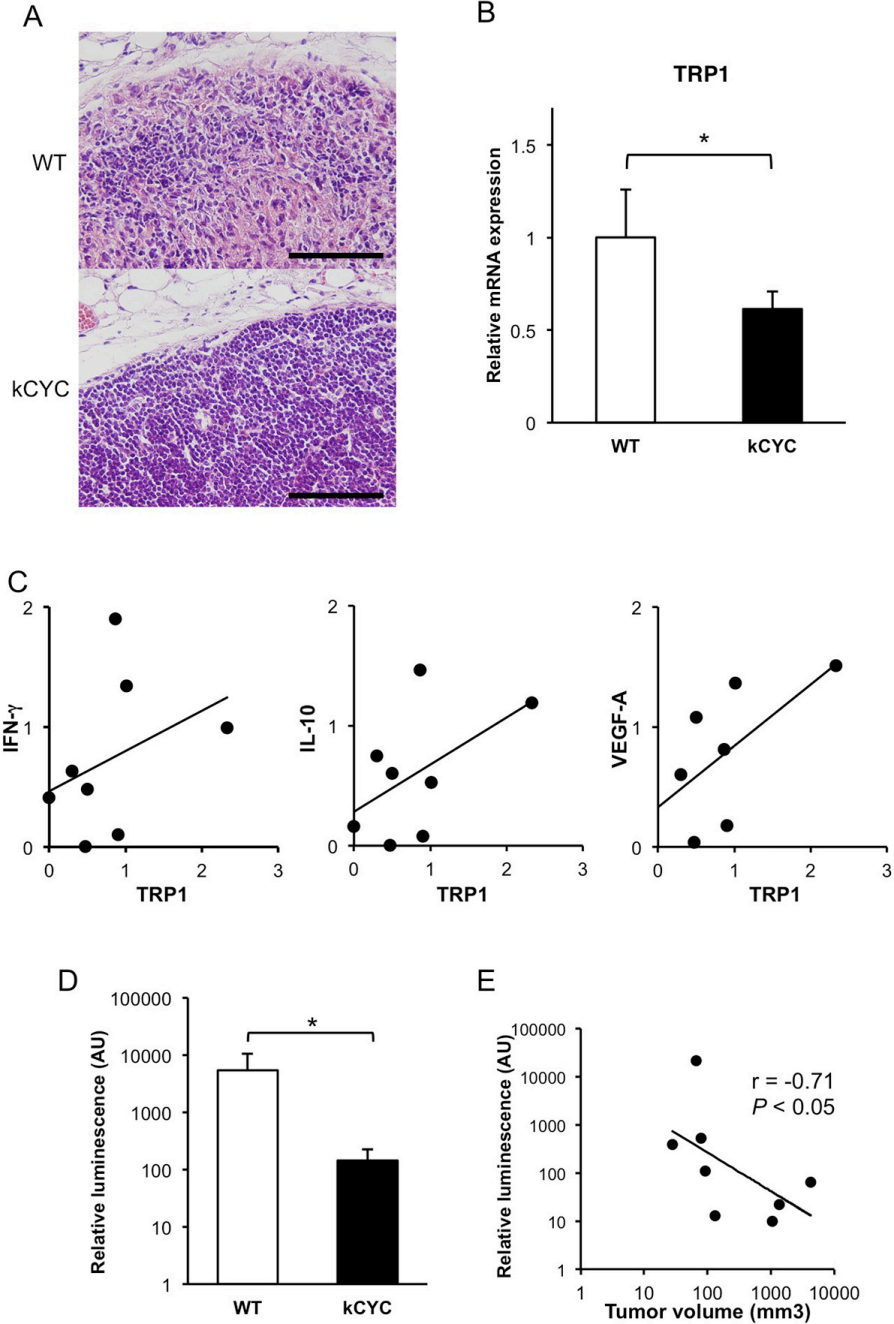
Less frequent tumor metastasis and decreased levels of tumor antigens in draining LNs of *kCYC* mice compared to wild-type (WT) mice **A.** Draining LNs were harvested 14 days following subcutaneous injection of B16 melanoma cells and stained with hematoxylin and eosin. Scale bars indicate 100 μm. Representative pictures were taken out of 6 mice in each group. **B.** Messenger RNA expression of tyrosinase-related protein 1(TRP1) was measured by quantitative PCR. Values represent means + SEM (*p < 0.05). Results were obtained from 6 mice in each group. **C.** Correlation between expression levels of TRP1 and those of interferon (IFN)-γ, IL-10, and vascular endothelial growth factor (VEGF)-A. There was a significant correlation between TRP1 expression and VEGF-A expression (*r* = 0.68, *p* < 0.05). **D.** Relative luminescence transported into draining LNs was quantified with a luminometer. Values represent means + SEM (*p < 0.05). Results were obtained from 4 mice in each group. **E.** Correlation between relative luminescence value and tumor volume was examined. The line indicates an approximate curve (*r* = −0.71, *p* < 0.05). Results were obtained from 8 mice.

Draining LNs were examined histologically to assess for metastasis of subcutaneously injected B16 melanoma cells. On day 14, melanoma cells infiltrated LNs and the structures of LNs were altered in some of WT mice, while metastases in LNs of *kCYC* mice were less prominent (Figure 3A). Indeed, the frequency of metastasis into draining LNs was significantly lower in kCYC mice (20%) compared to WT mice (83%; *p* < 0.05). Tyrosinase-related protein (TRP) 1, a representative melanocyte/melanoma-specific marker, was measured by quantitative PCR to semi-quantify melanoma metastases in LN [29]. TRP1 mRNA expression levels were significantly decreased in *kCYC* mice compared to WT mice (Figure 3B, *p* < 0.05). Messenger RNA expression levels of TRP1 tended to correlate with those of IFN-γ and IL-10 (Figure 3C, *r* = 0.43 and 0.18, respectively), although there was no statistical significance; there was a significant correlation between TRP1 expression and VEGF-A expression in draining LNs (Figure 3C, *r* = 0.68, *p* < 0.05). These data show that regional spread of melanoma cells and the subsequent cytokine expression in cancer-affected tissues are reduced in the setting of lymphatic dysfunction.

Since minimal levels of TRP1 mRNA originating from skin melanocytes can be detected within draining LNs even without melanoma metastases, we next utilized luciferase activity within LNs following skin injection of melanoma cells genetically engineered to express luciferase. Relative luciferase activity was significantly lower in draining LNs of *kCYC* mice compared to WT mice (Figure 3D, *p* < 0.05). Interestingly, primary tumor volumes in skin of WT and *kCYC* mice inversely correlated with relative luminescence values in draining LNs (Figure 3E, *r* = −0.71, *p* < 0.05). Thus, fewer tumor cells migrated to draining LNs in the setting of lymphatic dysfunction; this, in turn, may have caused greater primary tumor cell growth because of impaired tumor immunity, which is typically generated within LNs.

### Presentation of tumor antigens is attenuated in draining LNs and spleen of *kCYC* mice

**Figure 4:**
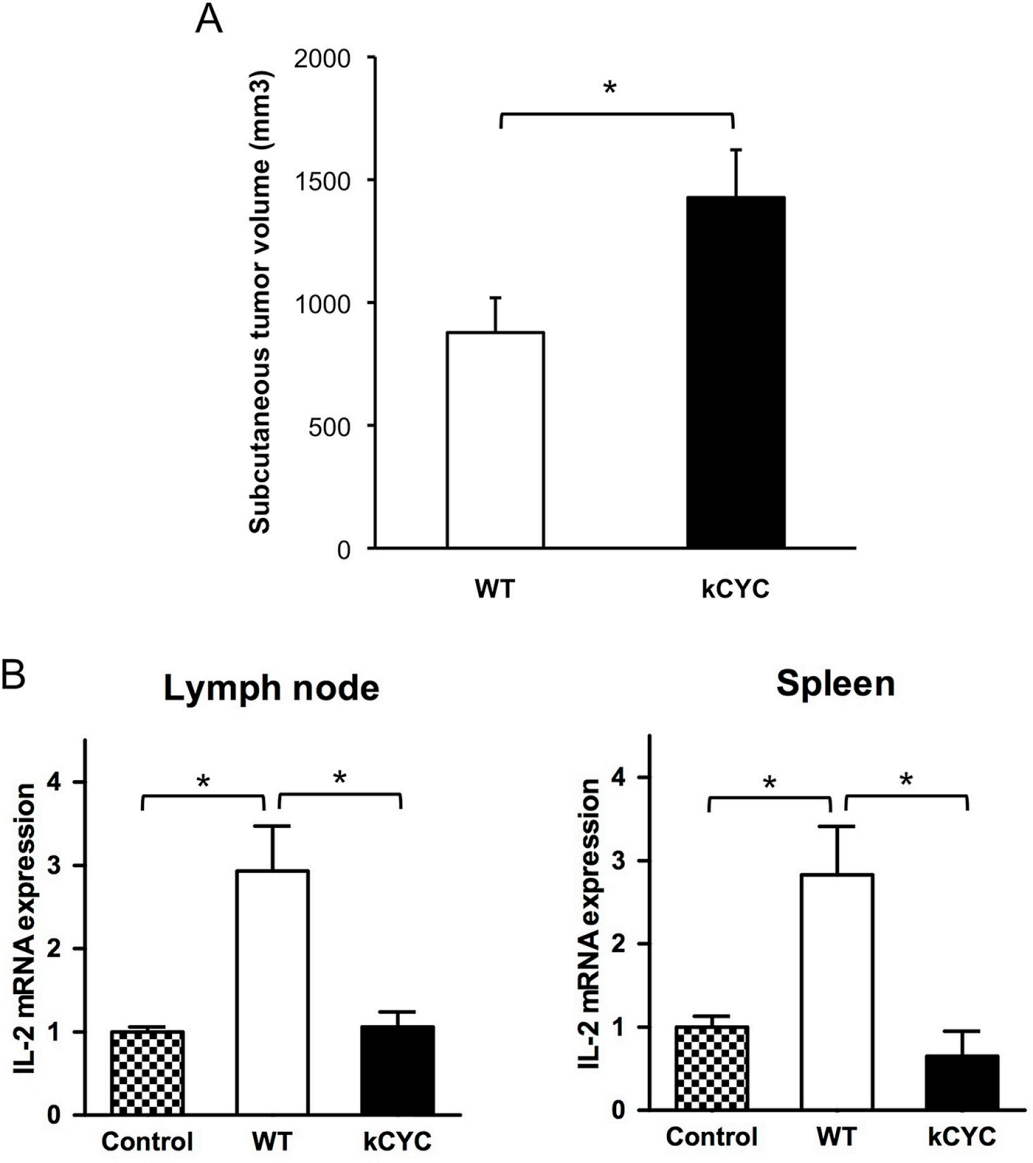
Presentation of tumor antigen is attenuated in *kCYC* mice **A.** Tumor volumes were measured 14 days following subcutaneous injection of MO4 melanoma cells into *kCYC* or wild-type (WT) mice. Values represent means + SEM (*p < 0.05). **B.** LNs or spleen were harvested from *kCYC* and WT mice receiving MO4 melanoma cells and cell suspensions were then co-cultured with RF33.70 cells for 24 hours. IL-2 mRNA expression in cultured cells was examined by quantitative PCR. RF33.70 cells cultured with cell suspensions derived from non-immunized mice served as a negative control. Values represent means + SEM (*p < 0.05). Results were obtained from 8 mice in each group.

To test the hypothesis that dysregulated lymphatic flow causes impaired tumor immunity, we examined antigen presentation of melanoma cells in draining LNs and spleen of *kCYC* mice. After injecting MO4 melanoma cells expressing OVA into *kCYC* and WT mice, CD11c^+^ DCs were isolated from draining LNs and spleen and cultured with RF33.70 cells, which react specifically with OVA to produce IL-2. We first confirmed that primary MO4 tumor growth was significantly enhanced in *kCYC* mice compared to WT mice (Figure 4A, *p* < 0.05), which was consistent with B16 and EL-4 tumorigenesis as shown in Figures 1A and 2A. IL-2 expression by RF33.70 cells was significantly increased when co-cultured with CD11c^+^ DCs from draining LNs in WT mice (Figure 4B, *p* < 0.05), but not elevated when CD11c^+^ DCs were derived from *kCYC* mice. Similar results were obtained when CD11c^+^ DCs were derived from spleen (Figure 4B). Thus, presentation of tumor antigens by DCs isolated from draining LNs and spleen was impaired in the setting of lymphatic dysfunction.

### Cytotoxic activity of CD8^+^ T cells in draining LNs is decreased in *kCYC* mice bearing B16 melanoma

**Figure 5:**
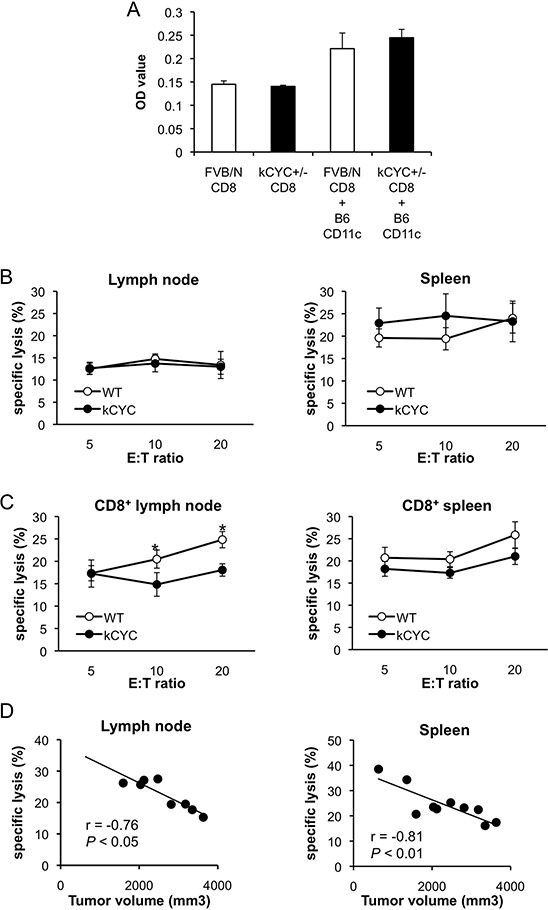
Cytotoxic activity of CD8^+^ T cells in draining LNs is decreased in *kCYC* mice bearing B16 melanoma cells **A.** CD8^+^ T cells derived from *kCYC*^+/–^ (FVB/N background) or wild-type FVB/N mice were co-cultured with CD11c^+^ cells derived from B6 mice. Proliferation of CD8^+^ T cells was examined with a Cell Proliferation ELISA, BrdU kit. **B.** Cell suspensions were prepared from draining LNs and spleen 14 days following subcutaneous injection of B16 melanoma cells. Non-purified cells were cultured with B16 melanoma cells at effector:target (E:T) ratios of 5:1, 10:1, and 20:1. Cytotoxic activity was assessed with two-color flow cytometry. All values represent means ± SEM. Results were obtained from 4 mice in each group. **C.** Cytotoxic responses directed against B16 melanoma by purified CD8^+^ LN cells or splenocytes from kCYC and wild-type (WT) mice were examined (*p* < 0.05). **D.** Correlation between tumor lytic rate at E:T ratios of 20:1 and tumor volumes were examined. The line indicates approximate curves (*r* = −0.76, *p* < 0.05, and *r* = −0.81, *p* < 0.01, respectively). Results were obtained from 8 and 10 mice, respectively.

After demonstrating that presentation of tumor antigens, which is critical for establishing adaptive immunity against tumor, was abrogated in *kCYC* mice, we next examined cytotoxic T cells responses in these mice. First, proliferation of CD8^+^ T cells isolated from *kCYC^+/–^* mice was assessed to determine whether *k-cyclin* gene expression affected T cell function. There were no differences in proliferation of CD8^+^ T cells between *kCYC^+/–^* (FVB/N background) and WT siblings when co-cultured with CD11c^+^ DC derived from C57BL/6 (B6) mice (Figure 5A). Tumor-draining LNs and spleen were harvested 14 days after subcutaneous injection of B16 melanoma cells and cell suspensions from these organs were tested for lytic activity against the tumor cells. Bulk LN cells obtained from *kCYC* and WT mice induced similar levels of lysis (Figure 5B). Splenocytes collected from these two groups of mice also induced similar levels of lysis. By contrast, when CD8^+^ T cells were purified from draining LNs, *kCYC* mice showed significantly decreased killing activity compared to WT mice (Figure 5C, *p* < 0.05). Purified CD8^+^ T cells isolated from spleen of *kCYC* mice also tended to induce lower levels of lysis than those obtained from WT mice. Of interest, cutaneous tumor volumes on day 14 inversely correlated with lytic activity of CD8^+^ T cells in draining LNs and spleen at an E:T ratio of 20:1 (Figure 5D; *r* = −0.76, *p* < 0.05, and *r* = −0.81, *p* < 0.01, respectively). Thus, cytotoxic activity of CD8^+^ T cells in draining LNs was decreased in the setting of lymphatic dysfunction, which may have also contributed to primary tumor growth within skin.

### Tumor suppressive activity of CD8^+^ T cells derived from *kCYC* mice is reduced when adoptively transferred into naive WT mice

**Figure 6:**
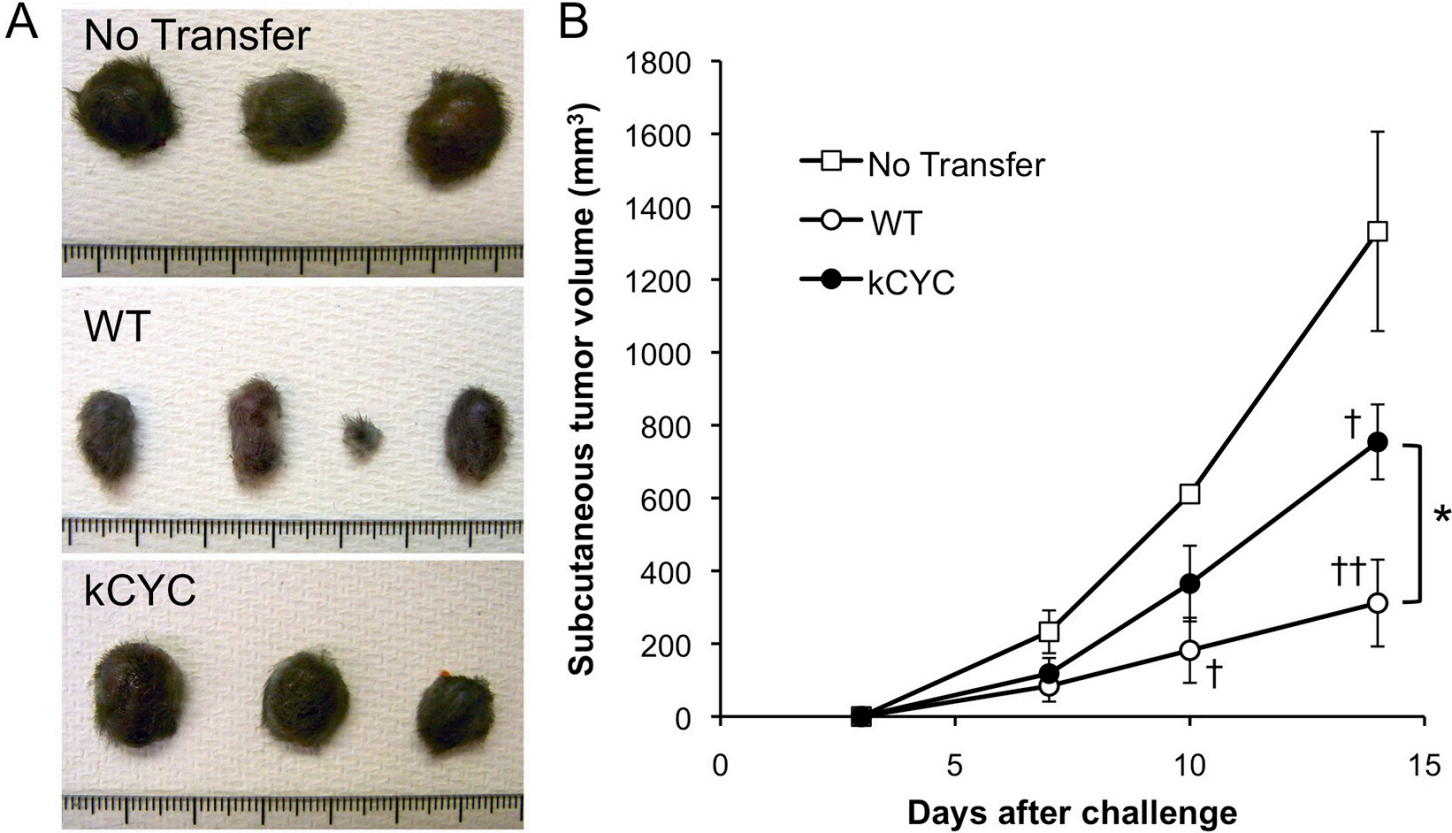
Tumor suppression of CD8^+^ T cells derived from *kCYC* mice is reduced when adoptively transferred to naive wild-type (WT) mice CD8^+^ T cells were isolated from *kCYC* and WT mice that were immunized with B16 cells. B16 cells were subcutaneously injected to naive WT mice one day after the adoptive transfer of CD8^+^ T cells. **A.** Representative images of the tumor. **B.** Tumor volumes were measured at the indicated time points following subcutaneous injection of B16 melanoma cells. Values represent means ± SEM (*p < 0.05). †*p* < 0.05, ‡*p* < 0.01 versus control group. Experiments were performed twice using 3 to 4 mice in each group.

Next, cytotoxic T cell activity of *kCYC* mice was directly examined by transferring CD8^+^ T cells into naive WT mice. Transfer of CD8^+^ T cells from draining LNs of immunized WT mice resulted in significant suppression of primary tumor growth on days 10 (*p* < 0.05) and 14 (*p* < 0.01; Figure 6A, 6B). Transfer of CD8^+^ T cells from immunized *kCYC* mice also induced suppression of tumor growth on day 14 (*p* < 0.05), but this was significantly lower than when cytotoxic cells were derived from WT mice (*p* < 0.05). Thus, CD8^+^ T cells isolated from tumor-bearing mice with lymphatic dysfunction showed impaired anti-tumor function when adoptively transferred into naive WT mice.

## DISCUSSION

Lymphatic vessels are critical for transporting tissue-resident DCs and other immune cells as well as interstitial fluid to draining LNs, establishing immunity against infectious agents and malignancy within tissues. On the other hand, they are also recognized as important conduits for tumor metastases [30]. Indeed, lymphangiogenesis within and around primary tumors has recently been linked with local cancer development and its dissemination. For example, VEGF-C or -D over-expression within breast cancer cells promotes intratumoral lymphangiogenesis, resulting in significantly enhanced metastasis to regional LNs and lungs [30, 31]. Increased lymphatic drainage has also been positively correlated with LN metastasis in breast cancer [32]. In addition, high central tumor interstitial fluid pressure was found to cause the development of pulmonary and LN metastases from melanoma [33]. Consistent with these published findings, melanoma and lymphoma metastasis to draining LN were reduced in mice with lymphatic dysfunction (Figure 3A).

Prior investigations on the dynamic role of lymphatic transport on tumor immunity using model mice have been limited. When melanoma cells were transplanted to the hind limbs of mice with lymphedema (created by radiation treatment and surgical division of the superficial and deep lymphatics), those mice frequently developed in-transit metastasis [13]. This finding is consistent with our results that primary cutaneous growth of either B16 melanoma or EL-4 lymphoma cells is augmented in mice with lymphatic dysfunction (Figures 1A and 2A). In another study, B16 melanoma cells were implanted into the footpads of mice, and tumor-draining popliteal LNs showed greatly increased lymphatic sinuses and accumulation of lymphocytes before apparent metastasis, indicating that primary tumors induce these alterations at a distance [14]. In our current study, cytokine expression in draining LNs was reduced in mice with lymphatic dysfunction (Figures 1B and 2B), suggesting that tumor-derived stimuli that provoke inflammatory tissue reactions did not reach draining LNs in our lymphedematous mice. Although it has been reported that IL-10 mediates anti-inflammatory responses [34], IL-10 could be produced by activated cells involved in a host antitumor reactions. Thus, it could produce a pro-inflammatory as opposed to an anti-inflammatory response to cancer [35]. In draining LNs of *kCYC* mice, there seemed to be no stimuli causing either inflammatory or immunoregulatory reactions. Decreased expression of VEGF-A, which mediates angiogenesis and drives inflammatory process, in *kCYC* mice also supports this assertion. TGF-β and IL-17A expression levels were not significantly different between *kCYC* and WT mice (Figures 1B and 2B), suggesting that overall decreased cytokine expression was specific to tumor immune responses. We also detected a significant correlation between expression levels of TRP1 and those of VEGF-A (Figure 3C), suggesting that the inflammatory process was induced by the presence of tumor cells in the melanoma-affected LNs.

We previously showed that lymphatic retention in *kCYC* mice almost completely blocked migration of skin DCs to draining LNs following epicutaneous sensitization [15]. This latter finding is consistent with decreased tumor antigen presentation in draining LNs as shown here (Figures 3B and 4B). *K14-VEGFR-3-Ig* mice, which showed defective lymphatic growth only in the skin, have also been used to assess the role of lymphatics in adaptive immune system [16, 17]. After topical treatment with a tumor initiator and repeated promoter applications, these mice developed significantly fewer squamous cell tumors compared with WT mice [17], suggesting an important role of VEGF-C/D in shaping the inflammatory tumor microenvironment that regulates early tumor progression. B16 melanoma cells expressing VEGF-C promoted host immune tolerance by activating lymphatic endothelial cells, which cross-presented tumor antigen to CD8^+^ T cells [18]. Although VEGF-C enhances transport to the draining LNs and antigen exposure to the adaptive immune system, it can have negative effect on tumor immunity by activating tumor-surrounding cells, inducing aberrant lymphangiogenesis, and promoting immune tolerance.

Interestingly, we showed that both the amount of tumor antigen (i.e., luciferase activity) and cytotoxic activity of CD8^+^ T cells in draining LNs inversely correlated with primary tumor volumes (Figures 3D and 5D). These results suggest that augmented tumor growth in *kCYC* mice did not represent a mere accumulation of tumor cells at inoculation sites, but resulted from impaired tumor immunity secondary to reduced transport of tumor-associated antigens and antigen presentation. Impaired anti-tumor function of CD8^+^ T cells from tumor-bearing *kCYC* mice adoptively transferred to naive WT mice (Figure 6) further supports this concept.

Tumor immunity is mediated by both the innate immune system, such as NK cells [2], and cells of adaptive immunity, such as DCs [3] and CD8^+^ T cells [4]. Cytotoxicity assays using whole cell populations derived from draining LNs and spleen directed against B16 melanoma cells showed no significant differences between WT and *kCYC* mice (Figure 5B). On the other hand, CD8^+^ T cells derived from tumor-immunized *kCYC* mice showed reduced cytotoxicity *in vitro* and *in vivo* (Figures 5C and 6). These results suggest that whole cell populations derived from LNs and spleen contained immune cells that target tumor cells independently of antigen presentation, such as NK cells, which recognize MHC-I molecules and kill target cells in the absence of antigen-presenting cells [36]. Although further study is needed to rule out the effects of other cell populations such as regulatory T cells and a unique tissue microenvironment in *kCYC* mice, we have definitively shown that lymphatic transport plays an essential role in generating tumor-specific immune responses mediated by DCs and CD8^+^ T cells.

Treatments for cancer include surgery, chemotherapy, radiation therapy, and combinations of these modalities. Since most types of cancer initially metastasize into draining LNs via lymphatics prior to systemic dissemination, regional LNs in cancer patients are often surgically removed. Our study, however, suggests that draining LNs are the “gatekeeper” of tumor immunity, and that removal of these organs could result in a poor prognosis. Indeed, elective LN dissection in melanoma of the limbs is not recommended, since it does not improve survival as shown randomized controlled trials [37–40]. Although lymphangiogenesis and vessel dilation are thought to increase delivery of lymph fluid and cells to draining LNs, decreased drainage has also been observed surrounding some cancers [41, 42]. Based on our findings here, we suggest that immunotherapeutic strategies for cancer should be pursued that allow for efficient delivery of tumor antigens to LNs while preventing cell metastases to these organs.

In summary, we revealed that primary tumor growth at the site of inoculation was augmented in the setting of severe lymphatic dysfunction. Cytokine expression in draining LNs was significantly attenuated. Moreover, decreased levels of tumor-associated antigens were detected in draining LNs along with impaired antigen presentation. Furthermore, CD8^+^ T cells in draining LNs derived from tumor-bearing mice with lymphatic dysfunction showed significantly decreased cytotoxic activity *in vitro* and decreased functional activity *in vivo*. Taken together, these findings suggest that lymphatic transport plays an essential role in generating tumor-specific immune responses mediated by DCs and CD8^+^ T cells.

## MATERIALS AND METHODS

### Mice

FVB/N wild-type mice were purchased from Clea Japan, Inc. (Tokyo, Japan). *kCYC^+/–^* mice (FVB/N background) were generated as previously described [23]. B6 mice were purchased from Jackson Labs (Bar Harbor, ME, USA). *kCYC^+/–^* mice were crossbred with B6 mice and F1 *kCYC^+/–^* mice (*kCYC* mice) and F1 WT mice were used in this study, unless otherwise mentioned. All mice were free of pathogenic bacteria and viruses. All experiments were performed using mice between 7 and 15 weeks of age. The Animal Committee of National Center for Global Health and Medicine approved all studies and procedures. Oligonucleotide primers used to genotype mice were as follows: *kCYC* forward: 5′-CTTCTGGATCCCACGCTATG-3′, *kCYC* reverse: 5′-TCTGTTCGCCACGCCAACTT-3′.

### Cells

B16/F1 melanoma cells that had been retrovirally transduced with luciferase cDNA were previously described as pLNCX2-B16 [43] and were termed “B16” cells herein for greater clarity. B16 cells and the mouse lymphoma cell line, EL-4, were kindly donated by Dr Sam T. Hwang (Medical College of Wisconsin, Milwaukee, WI). MO4, generated by the transfection of C57BL/6-derived melanoma B16 with the pAc-neo-OVA plasmid [44], and the T cell hybridoma, RF33.70, recognizing OVA_257–264_ in the context of Kb [45], were kindly donated by Dr. K. Lock (University of Massachusetts Medical Center, Boston, MA). B16 and EL-4 cells were maintained in Dulbecco's modified Eagle's medium (Sigma-Aldrich, St Louis, MO, USA) with 10% FBS and supplements (penicillin G sodium, streptomycin sulphate, and amphotericin B) at 37°C in 5% CO2. MO4 and RF33.70 cells were grown in RPMI 1640 (Millipore, Billerica, MA) with 10% FBS, supplements, and G418 sulphate (Calbiochem, San Diego, USA).

### Primary cutaneous tumor growth

B16 cells (5 × 10^5^) in 100 μl of phosphate-buffered saline (PBS) were injected subcutaneously into the shaved lateral flank of anaesthetized mice. The size of primary tumor was measured on days 3, 7, 10, and 14. The tumor volume was calculated using the equation: V = π(L1 × L2^2^)/6, where V = volume (mm^3^), L1 = longest diameter (mm), and L2 = shortest diameter (mm).

### Histologic examination

Tumor-draining LN was harvested 14 days after injection of B16 cells. Specimens were fixed in 3.5% paraformaldehyde and paraffin-embedded. Paraffin sections were stained with H&E to evaluate tumor metastases.

### RNA isolation and quantitative reverse transcriptase (RT)-PCR

Total RNA was extracted from frozen tissue samples using RNeasy Fibrous Tissue Mini Kit (QIAGEN, Hilden, Germany) and was subsequently reverse-transcribed to cDNA using the iScript cDNA synthesis kit (Bio-Rad, Hercules, CA, USA), according to the manufacturers’ protocols. Real-time RT-PCR was performed using the Fast SYBR^®^ Green Master Mix (Applied Biosystems, Foster City, CA, USA) on StepOnePlus™ Real-Time PCR System (Applied Biosystems), according to the manufacturers’ instructions. Primers for IL-18 were purchased from Applied Biosystems. Other primers for mouse genes were as follows: IFN-γ forward, 5′-TCA AGT GGC ATA GAT GTG GAA GAA-3′ and reverse, 5′-TGG CTC TGC AGG ATT TTC ATG-3′; TNF-α forward, 5′-CCA CCA CGC TCT TCT GTC TAG-3′ and reverse, 5′- AGG GTC TGG GCC ATA GAA CT-3′; TGFβ1 forward, 5′-TTG CTT CAG CTC CAC AGA GA-3′ and reverse, 5′-TGG TTG TAG AGG GCA AGG AC-3′; IL-2 forward, 5′-TGA GCA GGA TGG AGA ATT ACA GG-3′ and reverse, 5′- GTC CAA GTT CAT CTT CTA GGC AC-3′; IL-10 forward, 5′-TTT GAA TTC CCT GGG TGA GAA-3′ and reverse, 5′-ACA GGG GAG AAA TCC ATG ACA-3′; IL-17A forward, 5′- CTC CAG AAG GCC CTC AGA CTA C-3′ and reverse, 5′- AGC TTT CCC TCC GCA TTG ACA CAG-3′; VEGF-A forward, 5′-CTG CTG TAC CTC CAC CAT GC-3′ and reverse 5′-TCA CTT CAT GGG ACT TCT GCT CT-3′; and glyceraldehyde-3-phosphate dehydrogenase (GAPDH) forward, 5′-CGT GTT CCT ACC CCC AAT GT-3′ and reverse, 5′-TGT CAT CAT ACT TGG CAG GTT TCT-3′. TRP1 gene was selected as a representative melanocyte/melanoma-specific marker to identify melanoma cells that had metastasized to draining LNs [29, 46, 47]. The primer set for TRP1 was forward, 5′- GAA AAT ATG ACC CTG CTG TTC GA-3′ and reverse, 5′- TTG TCC TCC CGT TCC ATT CA-3′. Relative expression of PCR products was determined using the Δ*C*_T_ method [48]. Briefly, each set of samples was normalized using the difference in threshold cycle (*C*_T_) between the target gene and housekeeping gene (GAPDH): Δ*C*_T_=(*C*_T target gene_ − *C*_T GAPDH_). Each sample was examined in duplicate and the mean *C*_T_ was used in the equation.

### Luciferase assay

LNs and spleen were harvested from mice 14 days after the subcutaneous injection of B16 cells. Collected organs were minced with surgical scissors and lysed in Glo lysis buffer (Promega, Madison, WI). After centrifugation, the supernatant was collected and luminescence was analyzed with the Luciferase Assay System (Promega), according to the manufacturer's instructions. The lysate was mixed with the luciferase assay reagent and relative luminescence value was measured with the manual luminometer Luminescencer-PSN (ATTO, Tokyo, Japan).

### T cell hybridoma assay for detection of OVA

Tumor-draining LNs and spleen were harvested from mice 14 days after the subcutaneous injection of MO4 cells expressing OVA [44]. Single-cell suspensions were prepared and CD11c^+^ cells were isolated with CD11c MicroBeads (Miltenyi Biotec, CA, USA). CD11c^+^ cells (2 × 10^4^ cells) were co-cultured with RF33.70 (4 × 10^5^ cells) in 96-well flat-bottom culture plates for 24 hours. RF33.70 cells react specifically with OVA to produce IL-2 [49]. Total RNA was extracted with TRIzol (Invitrogen, Carlsbad, CA) and was subsequently reverse-transcribed to cDNA using iScript cDNA synthesis kit (Bio-Rad). IL-2 mRNA expression was quantified by real-time quantitative RT-PCR as described above.

### Mixed lymphocyte reaction

Axillary and inguinal LNs were harvested from *kCYC^+/–^* (FVB/N background), FVB/N, and B6 mice and single-cell suspensions were prepared. CD11c^+^ DCs and CD8^+^ T cells were isolated with MACS magnetic beads (Miltenyi Biotec). CD8^+^ T cells (3 × 10^4^ cells) derived from *kCYC^+/–^* (FVB/N background) or FVB/N mice were co-cultured with CD11c^+^ cells (3 × 10^4^ cells) derived from B6 mice in 96-well flat-bottom culture plates for 24 hours. Proliferation of CD8^+^ T cells was examined with the Cell Proliferation ELISA, BrdU (Roche Applied Science, Basel, Switzerland), according to the manufacturer's instructions.

### Cytotoxicity assay

Donor mice were injected with 5 × 10^5^ B16 cells in 100 μl of PBS subcutaneously into the lateral flank. Fourteen days after injections, mice were sacrificed. Spleen and draining inguinal LNs were harvested and single-cell suspensions were prepared. To assess antigen-specific T cell cytotoxic activity, CD8^+^ T cells were purified with the CD8a^+^ T Cell Isolation Kit II (Miltenyi Biotec) in some experiments, according to the manufacturer's instructions.

Cytotoxicity was assayed using a cell-mediated cytotoxicity kit (Molecular Probes, Eugene, OR, USA), according to the manufacturer's instructions. B16 target cells were incubated with a staining solution containing DiOC_18_ at 37°C. The stained target cells (1 × 10^6^ cells/ml) were re-suspended in complete culture medium and then mixed with LN cells or splenocytes to yield effector:target (E:T) ratios of 20:1, 10:1, and 5:1. A counterstaining solution containing propidium iodide to detect dead cells was added and the mixture was incubated at 37°C for 2 hours. To assess lytic activity, two-color flow cytometry was performed using a BD FACSVerse (BD Biosciences). The percentage of lysis was calculated by dividing the frequency of dead target cells by the total number of target cells.

### Adoptive transfer

Donor mice were inoculated with 5 × 10^5^ B16 cells in the left flank. Tumor-draining LNs were harvested 14 days after primary tumor inoculation. 5 × 10^5^ CD8^+^ T cells purified from *kCYC* or WT mice bearing B16 melanoma were injected intravenously into naive WT mice. The same amount of phosphate buffered saline was intravenously injected into WT mice as a negative control. All mice were challenged with 5 × 10^5^ B16 cells 1 day after adoptive transfer, and tumor growth was monitored for 14 days.

### Statistics

All data are shown as mean values ± standard error of the mean (SEM). Statistical analysis between two groups was performed using the Mann-Whitney's *U*-test or the Chi-square test. Correlation coefficients were determined using the Spearman's rank correlation test. *P*-values of < 0.05 were considered statistically significant.
